# Prognostic Significance of Nuclear Phospho-ATM Expression in Melanoma

**DOI:** 10.1371/journal.pone.0134678

**Published:** 2015-08-14

**Authors:** Madhuri Bhandaru, Magdalena Martinka, Kevin J. McElwee, Anand Rotte

**Affiliations:** 1 Department of Dermatology and Skin Science, University of British Columbia, Vancouver, British Columbia, Canada; 2 Department of Pathology and Laboratory Medicine, University of British Columbia, Vancouver, British Columbia, Canada; 3 Replicel Inc, Vancouver, British Columbia, Canada; Florida International University, UNITED STATES

## Abstract

UV radiation induced genomic instability is one of the leading causes for melanoma. Phosphorylation of Ataxia Telangiectasia Mutated (ATM) is one of the initial events that follow DNA damage. Phospho-ATM (p-ATM) plays a key role in the activation of DNA repair and several oncogenic pathways as well as in the maintenance of genomic integrity. The present study was therefore performed to understand the significance of p-ATM in melanoma progression and to correlate it with patient prognosis. Tissue microarray and immunohistochemical analysis were employed to study the expression of p-ATM in melanoma patients. A total of 366 melanoma patients (230 primary melanoma and 136 metastatic melanoma) were used for the study. Chi-square test, Kaplan-Meier, univariate and multivariate Cox regression analysis were used to elucidate the prognostic significance of p-ATM expression. Results revealed that both loss of, and gain in, p-ATM expression were associated with progression of melanoma from normal nevi to metastatic melanoma. Patients whose samples showed negative or strong p-ATM staining had significantly worse 5-year survival compared to patients who had weak to moderate expression. Loss of p-ATM expression was associated with relatively better 5-year survival, but the corresponding 10-year survival curve almost overlapped with that of strong p-ATM expression. p-ATM expression was found to be an independent prognostic factor for 5-year but not for 10-year patient survival. In conclusion our findings show that loss of p-ATM expression and gain-in p-ATM expression are indicators of worse melanoma patient survival.

## Introduction

Ataxia telangiectasia mutated (ATM) is a protein kinase belonging to the superfamily of phosphatidylinositol 3-kinase related kinases (PIKKs), is well known for its role in activation of DNA damage response pathways following DNA double strand breaks (DSBs) [1–4]. ATM reportedly responds to DSBs, by autophosphorylation at Ser-1981, leading to dissociation of inactive multimeric ATM to form active monomers, which allows it to associate with MRN complexes (MRE 11, RAD50 and NBS1 complex) and phosphorylate its downstream regulators of DNA repair such as histone H2AX, MDC1, 53BP1 and BRCA1 [1–3]. In addition to canonical responses to DSBs, ATM has also been shown to regulate the repair of single strand breaks (SSBs) and nucleotide excision repair [5–9].

Functional loss of ATM, caused by hereditary mutations in the gene predisposes individuals to Ataxia Telangiectasia (A-T); an autosomal recessive disorder characterized by neurodegeneration, immune deficiency, hypersensitivity to ionizing radiation and increased frequency of cancers [10–11]. Mutations in the ATM gene have been reported in several types of cancers including familial pancreatic, breast, ovarian, colorectal, haematologic cancers and in lung cancers illustrating the significance of ATM in cancer pathogenesis [12–17]. Studies in tumor samples from pancreatic, breast and gastric cancer patients revealed a correlation between loss of ATM expression, disease progression and poor prognosis, indicating its tumor suppressing nature [18–21]. Interestingly, tissue microarray analysis of samples from endometrial cancer patients showed a correlation between ATM positivity, disease progression and increased probability of recurrence, pointing towards the oncogenic nature of ATM [22]. Similarly, analysis of phosphorylated ATM (p-ATM) expression in cervical cancer patients revealed an association between high nuclear p-ATM expression in tumor samples and locoregional disease free survival, as well as poor response to chemotherapy [23].

ATM is known to activate oncogenic signalling cascades with its targets including kinases like Akt/PKB, and transcription factors like Nuclear Factor Kappa Beta (NFκB), and Hypoxia Inducible Factor-1α (HIF1α) [1]. In line with the results from endometrial cancer patients, analysis of ATM expression (mRNA and protein) in melanoma reported an upregulation of ATM in nodular malignant melanoma samples [24]. However, the sample size of the study was small with less than twenty five cases and the study was unable to clarify the association between ATM expression and prognosis. Moreover, the study did not analyze p-ATM expression in patients. Considering the significance of phosphorylation in monomerization and activation of ATM, there is a need for a clear analysis on the correlation between p-ATM expression and melanoma patient survival [25–31].

ATM is a large protein with molecular size of approximately 350 kD, and contains PI3K-like, FAT and FATC domains towards the c-terminal [1–2, 32]. Several sites of phosphorylation on ATM have been identified, including ser-367, ser-784, ser-1403, ser-1893, ser-1981, ser-2996, & Lys-3016 (acetylation), and reported to regulate the activity of ATM [1]. Among them phosphorylation at ser-1981 is most commonly studied and also reported to be involved in oxidative stress induced autophosphorylation [33]. Since, UV-induced pyramidine dimers, 6–4 photo products as well as 8-oxoguanine lesions on DNA are considered as the main initiating factors of melanoma, and ser-1981 phosphorylated ATM is considered to be involved in the subsequent repair of DNA, we asked if ser-1981 phosphorylation of ATM is associated with melanoma progression and prognosis [1, 5–9, 34]. Using tissue samples collected from melanoma patients, we analyzed phospho-ATM (ser-1981) expression in melanoma patients and studied the correlation with disease progression and patient survival.

## Methods

### Ethics Statement

The use of human skin tissues and the waiver of patient consent in this study were approved by the Clinical Research Ethics Board of the University of British Columbia [27]. Patient information was anonymized and de-identified prior to analysis. The study was conducted according to the principles expressed in the Declaration of Helsinki.

### Patient specimens and tissue microarray construction

The collection of patient specimens and the construction of the tissue microarray (TMA) have been previously described [35]. We used patient data collected between 1990 and 2009. Of the 748 patients specimens collected, 425 biopsies including 366 melanoma and 59 cases of nevi (27 normal nevi and 32 dysplastic nevi) could be evaluated for p-ATM staining in this study, due to loss of biopsy cores or insufficient tumor cells present in the cores. The criteria for patient exclusion and inclusion and the demographic characteristics of melanoma patients are detailed in S1 Table. All specimens were obtained from the archives of the Department of Pathology, Vancouver General Hospital.

The most representative tumor area in each biopsy was carefully selected and marked on the hematoxylin and eosin stained slides and the TMAs were assembled using a tissue-array instrument (Beecher Instruments, Silver Spring, MD). Tissue cores of 0.6-mm thickness were taken in duplicate from each biopsy. Using a Leica microtome, multiple 4 μM sections were cut and transferred to adhesive-coated slides using regular histological procedures. Tissue samples from melanoma and benign nevus were built into each TMA slide as positive and negative controls. One section from each TMA was routinely stained with hematoxylin and eosin whereas the remaining sections were stored at room temperature for subsequent immunohistochemical staining.

### Immunohistochemistry

Tissue microarray (TMA) slides were dewaxed at 55°C for 20 min followed by three 5 min washes with xylene. The tissues were then rehydrated by washing the slides for 5 min each with 100%, 95%, 80% ethanol and finally with distilled water. The slides were heated to 95°C for 30 min in 10 mmol/L sodium citrate (pH 6.0) for antigen retrieval and then treated with 3% hydrogen peroxide for 1 hour to block endogenous peroxidase activity. After blocking the slides with the universal blocking serum (Dako Diagnostics, Carpinteria, CA, USA), the sections were incubated overnight with mouse anti-p-ATM (ser 1981) antibody (1:50 dilution; Biolegend, USA) at 4°C. The sections were incubated for 30 min with a biotin-labelled secondary antibody and then with streptavidin-peroxidase (Dako Diagnostics). The labelling was developed by treatment with 3,3’-diamino-benzidine substrate (Vector Laboratories, Burlington, Ontario, Canada) and with hematoxylin to counter-stain the nuclei. Negative controls were done by omitting the p-ATM antibody during the primary antibody incubation.

### Evaluation of immunostaining

The evaluation of p-ATM staining was done blindly by microscopic examination of the tissue sections by one dermatopathologist and two other observers simultaneously, using a multiple viewing microscope and a consensus was reached for the score of each core. p-ATM staining intensity was scored as 0+, 1+, 2+, 3+ whereas the percentage of p-ATM positive cells was scored as 1 (1–25%), 2 (26–50%), 3 (51–75%) and 4 (76–100%). In cases of score discrepancy between duplicated cores, the higher score from the two tissue cores was taken as the final score. The product of intensity and percentage was taken as the immunoreactive score (IRS) [36]. Based on the IRS, p-ATM staining in the tissue sections was categorized as negative (IRS 0), weak (IRS 1–4), moderate (IRS 6–8), or strong (IRS 9–12). The optimum cut-off values for the IRS were derived based on the IRS pattern in nevi and melanoma. The cut-off points were additionally confirmed using the X-tile software (Yale University) [37].

### Statistical analysis

Correlation between p-ATM and clinicopathologic parameters was evaluated by Chi-square test among patient subgroups. Survival time was calculated from the date of melanoma diagnosis to the date of death or last follow-up. The association between p-ATM expression and patient survival (overall and disease-specific survival) was evaluated by Kaplan-Meier analysis and log-rank test. Additionally, univariate and multivariate Cox proportional hazards regression models were preformed to estimate the crude hazard ratios (HRs) or adjusted HRs and their 95% confidential intervals (CIs). *P*-value <.05 was considered as statistically significant. All the statistical analyses were performed using SPSS version 16.0 (SPSS Inc, Chicago, IL) software.

## Results

### Increase in as well as loss of p-ATM expression in the nucleus is associated with melanoma progression

**Fig 1:**
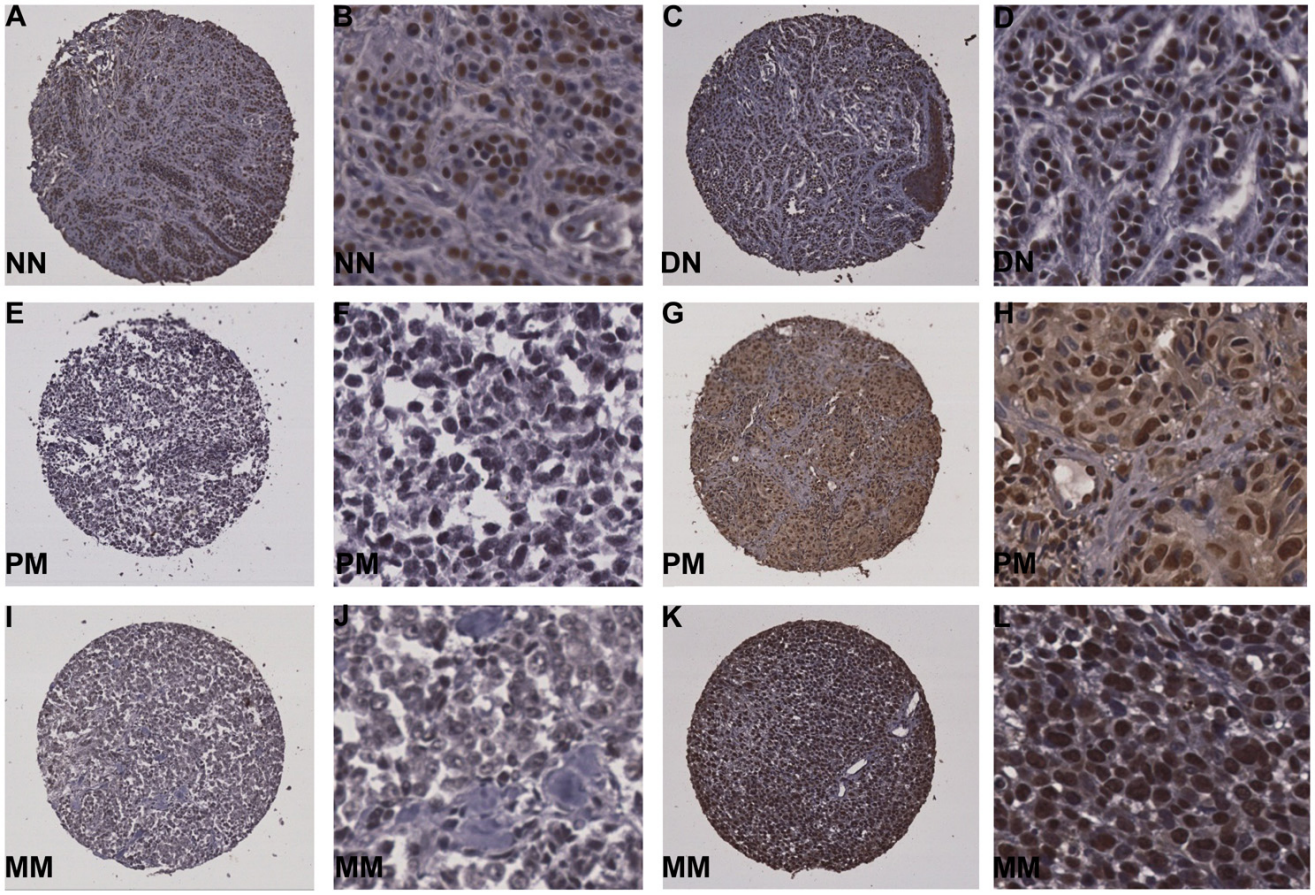
pATM expression in nuclei of melanoma samples. (A, B) Representative images of normal nevi (NN) and (C, D) dysplastic nevi (DN) with strong nuclear staining; (E-H) primary melanoma (PM) with negative (IRS-0, E & F) and strong (IRS 9–12, G & H) nuclear staining; (I-L) metastatic melanoma (MM) with negative (I & J) and strong (K & L) nuclear p-ATM staining.

As seen in Fig 1 and S1 Fig, p-ATM expression was mainly seen in the nucleus and therefore we evaluated the staining in the nuclei of the patient biopsies. IRS scores for the p-ATM expression in the normal nevi were in between 1 & 8 with none of the cores achieving a score of either 0 or 9 to 12, whereas, 12.5% cases of dysplastic nevi samples were scored as ‘negative’ (0) and 6.25% of cases received an IRS score of 9 to 12. The biopsies scored as negative increased to nearly 15% in primary melanoma cases and to 20.6% in metastatic melanoma cases. Similarly, the percentage of cases receiving scores >9, was increased to 11.7% in primary melanoma and 19.1% in metastatic melanoma. Therefore based on the IRS scoring patterns, we classified p-ATM expression as negative (IRS-0), weak to moderate (IRS 1–8), and strong (IRS 9–12), and analyzed the p-ATM expression in the patient biopsies using chi-square analysis.

**Fig 2:**
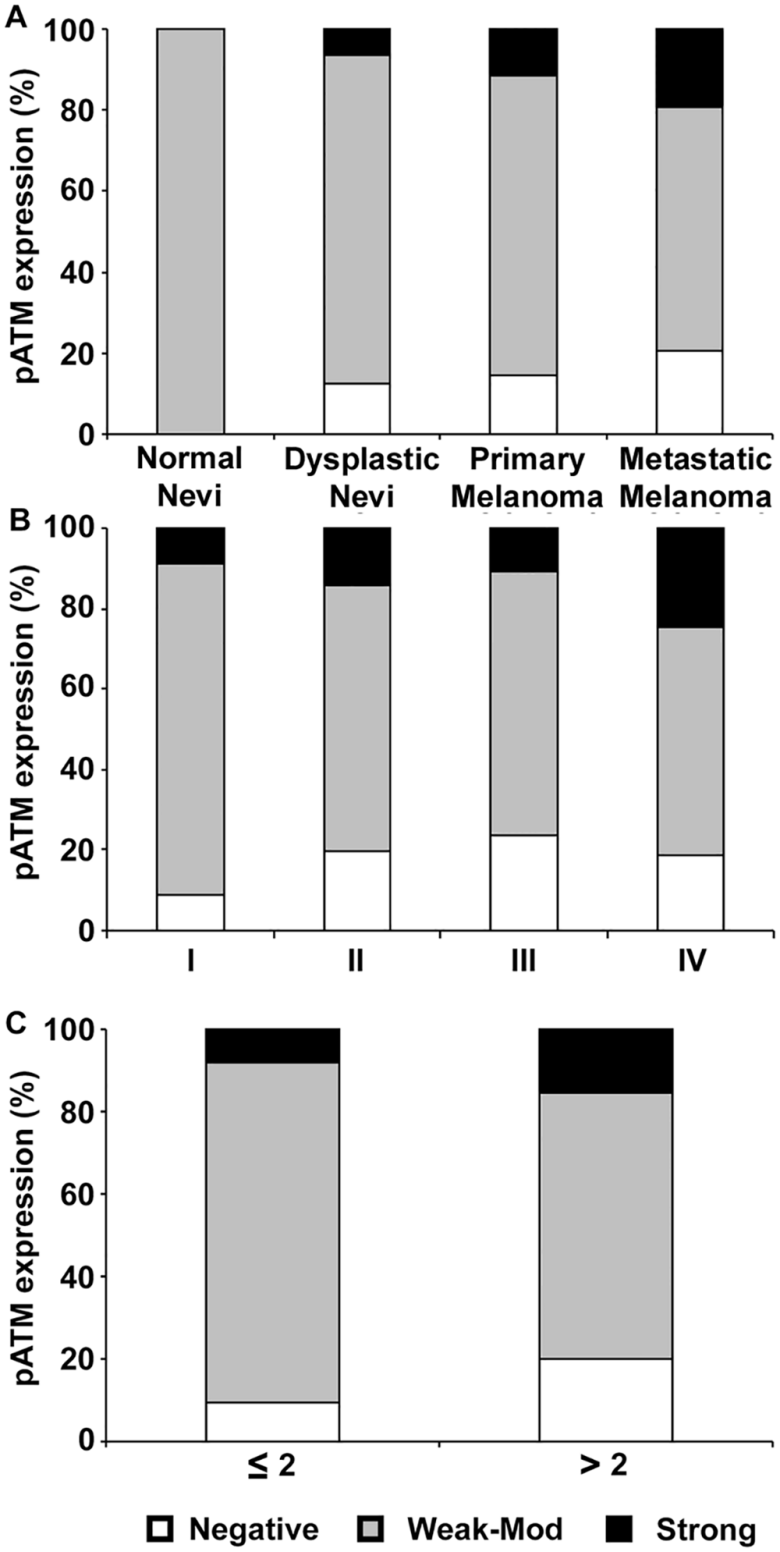
Correlation between pATM expression and melanoma progression. (A) Percentage of negative (IRS 0) and strong pATM (IRS 9–12) staining in nuclei is significantly increased from normal and dysplastic nevi to melanoma by χ^2^ test.(B) Loss of pATM expression as well as increase in pATM expression in the nucleus is significantly associated with melanoma progression by χ^2^ test. (C) Loss of pATM expression as well as increase in pATM expression in the nucleus is significantly associated with tumor thickness by χ^2^ test.

As shown in Fig 2A, cases of both negative and strong p-ATM expressions were increased from normal nevi to dysplastic nevi but the difference did not reach statistical significance (p = 0.0597) probably due to the small sample number. There also seemed to be a marginal, but not statistically significant, increase in the percentage of negative and strong p-ATM expressions from dysplastic nevi to primary melanoma (p = 0.592). The increase in negative and strong p-ATM expressions was obvious and statistically significant (p = 0.025) from primary melanoma to metastatic melanoma. In order to verify the association between p-ATM expression and melanoma progression, we divided the cases based on AJCC stage and analyzed the correlation between p-ATM and AJCC stage. As seen in Fig 2B, percentage of patients with negative p-ATM expression appeared to increase from stage I to stage III and then decreased from stage III to stage IV, though the differences between melanoma stages were not statistically significant. In contrast, there was no clear difference in strong p-ATM expression between AJCC stages I through III. Percentage of patients with strong p-ATM expression tended to increase from AJCC stage III to stage IV, but the difference was not statistically significant (Fig 2B).

### p-ATM expression and clinicopathologic characteristics

To further test if loss or increase of p-ATM expression was related to increase in tumor thickness, we classified our database on the basis of tumor thickness ≤ 2 mm or > 2 mm and analyzed the correlation. Chi square test analysis of the data revealed that biopsies from patients with larger tumors (> 2 mm) had significantly higher incidence of negative or strong p-ATM staining (p = 0.009, Fig 2C, Table 1). We continued with the analysis by dividing the data based on other clinicopathologic characteristics such as ulceration status, sun exposure and tumor subtype. We did not find any correlation between p-ATM expression and the rest of the demographic and clinicopathologic characteristics (Table 1).

**Table 1:** p-ATM staining and clinicopathological characteristics of 366 melanoma patients.

Variables	Nuclear p-ATM			
	Negative	Weak-to-Moderate	Strong	*p*-value*
**All Melanoma**				
Age				
≤ 60	31 (16.4%)	130 (68.8%)	28 (14.8%)	0.951
> 60	31 17.5%)	121 (68.4%)	25 (14.1%)	
Gender				
Male	35 (16.0%)	147 (67.1%)	37 (16.9%)	0.264
Female	27 (18.4%)	104 (70.7%)	16 (10.9%)	
AJCC stage				
I	9 (8.8%)	84 (82.4%)	9 (8.8%)	0.004
II	25 (19.5%)	85 (66.4%)	18 (14.1%)	
III	13 (23.6%)	36 (65.5%)	6 (10.9%)	
IV	15 (18.5%)	46 (56.8%)	20 (24.7%)	
Site				
Sun Protected	43 (39.5%)	194 (60.5%)	44 (60.5%)	0.211
Sun Exposed	19 (44.1%)	57 (55.9%)	9 (55.9%)	
**Primary Melanoma**				
Age				
≤ 60	15 (13.6%)	82 (74.5%)	13 (11.8%)	0.895
> 60	19 (15.8%)	87 (72.5%)	14 (11.7%)	
Gender				
Male	18 (15.5%)	92 (74.8%)	17 (9.7%)	0.681
Female	16 (14.2%)	77 (72.4%)	10 (13.4%)	
Tumor Thickness				
≤ 2	11 (9.6%)	94 (82.5%)	9 (7.9%)	0.009
> 2	23 (19.8%)	75 (64.7%)	18 (15.5%)	
Ulceration				
Absent	22 (12.4%)	136 (76.8%)	19 (10.7%)	0.094
Present	12 (22.6%)	33 (62.3%)	8 (15.1%)	
Subtype				
Acrolentigous	3(37.5%)	4 (50.0%)	1 (12.5%)	0.308
Lentigous	4 (11.8%)	26 (76.4%)	4 (11.8%)	
Nodular	7 (15.6%)	30 (66.7%)	8 (17.7%)	
Spindle cell	3(30.0%)	7 (70.0%)	0 (0.0%)	
Superficially spreading	8 (10.3%)	64 (82.1%)	6 (7.6%)	
Unspecified	9 (16.4%)	38 (69.1%)	8 (14.5%)	
**Metastatic Melanoma**				
Age				
≤ 60	16 (49.4%)	48 (50.6%)	15 (50.6%)	0.990
> 60	12 (43.5%)	34 (56.5%)	11 (56.5%)	
Gender				
Male	17 (18.5%)	55 (59.8%)	20 (21.7%)	0.438
Female	11 (25.0%)	27 (61.4%)	6 (13.6%)	

Sun-protected sites: trunk, arm, leg and feet; Sun-exposed sites: head and neck.

*- χ^2^ test.

DNA alkylating agents like dacarbazine and temozolomide are used in melanoma treatment [38–39]. DNA damage is known to phosphorylate ATM at serine-1981 and activate the kinase, so we explored p-ATM expression in patients treated with DNA damaging agents [1, 3, 40]. Based on the chemotherapeutic treatment information available in our database, we pooled 21 patients who were administered DNA damaging agents and analyzed p-ATM expression in them. As seen in S2 Fig, negative p-ATM expression appeared to be more frequent in patients treated with DNA damaging agents, but the correlation was not statistically significant (p = 0.328). In vitro studies attempting to sensitize melanoma cells to radiation and/or chemotherapy by inhibition of ATM demonstrated conflicting results. While ATM inhibition was not sufficient to sensitize melanoma cells to ionizing radiation, cells lacking ATM activity were found to be sensitive to temozolamide treatment [41–43]. We therefore analyzed the information from DNA damaging agents administered patients to check if the p-ATM expression correlated with treatment resistance. Interestingly, strong as well as negative p-ATM expression was found to be significantly associated with resistance to chemotherapy in melanoma patients (S3 Fig, p = 0.0194).

### Nuclear p-ATM expression and 5-year survival

**Fig 3:**
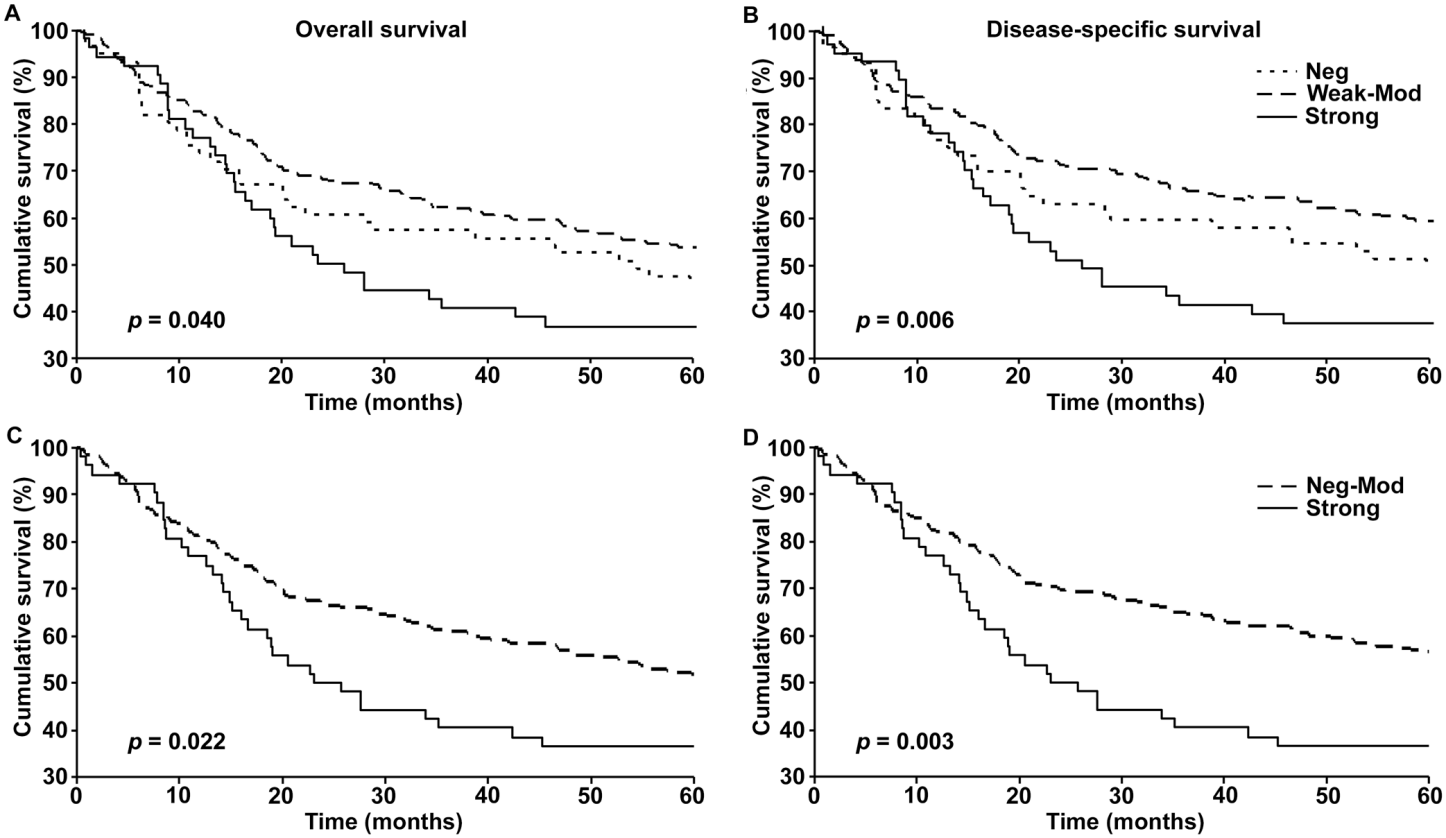
pATM expression and 5-year patient survival. Kaplan-Meier survival analyses of correlation between pATM expression and 5-year overall (left panels) and disease-specific (right panels) survival of melanoma patients classified as negative (Neg), weak-to-moderate (Weak-Mod) & strong (A and B), and as negative-to-moderate (Neg-Mod) & strong (C and D).

We then asked if the loss of or gain in p-ATM expression was associated with 5-year survival of melanoma patients. Kaplan-Meier analysis of patient survival revealed that patients with weak to moderate expression of p-ATM had significantly better overall (p = 0.04) and disease specific (p = 0.006) survival compared to patients with negative or strong p-ATM expression (Fig 3A and 3B). Of the three groups, patients with strong p-ATM expression had the worst prognosis; the prognosis of patients whose tissue samples were negative for p-ATM was comparatively better and the survival curve was found to be closer to the weak to moderate expression group (Fig 3A and 3B). We then combined the patients with negative and weak to moderate expression into one group and compared their survival with patients with strong expression only. As seen in Fig 3C and 3D, strong p-ATM was significantly associated with worse overall (p = 0.022) and disease specific (p = 0.003) survival. We then confirmed the association between p-ATM and patient survival, using univariate Cox regression analysis and found that patients with strong p-ATM expression had significantly worse survival compared to patients with negative to moderate expression of p-ATM (S2 Table).

### Nuclear p-ATM expression and 10-year survival

**Fig 4:**
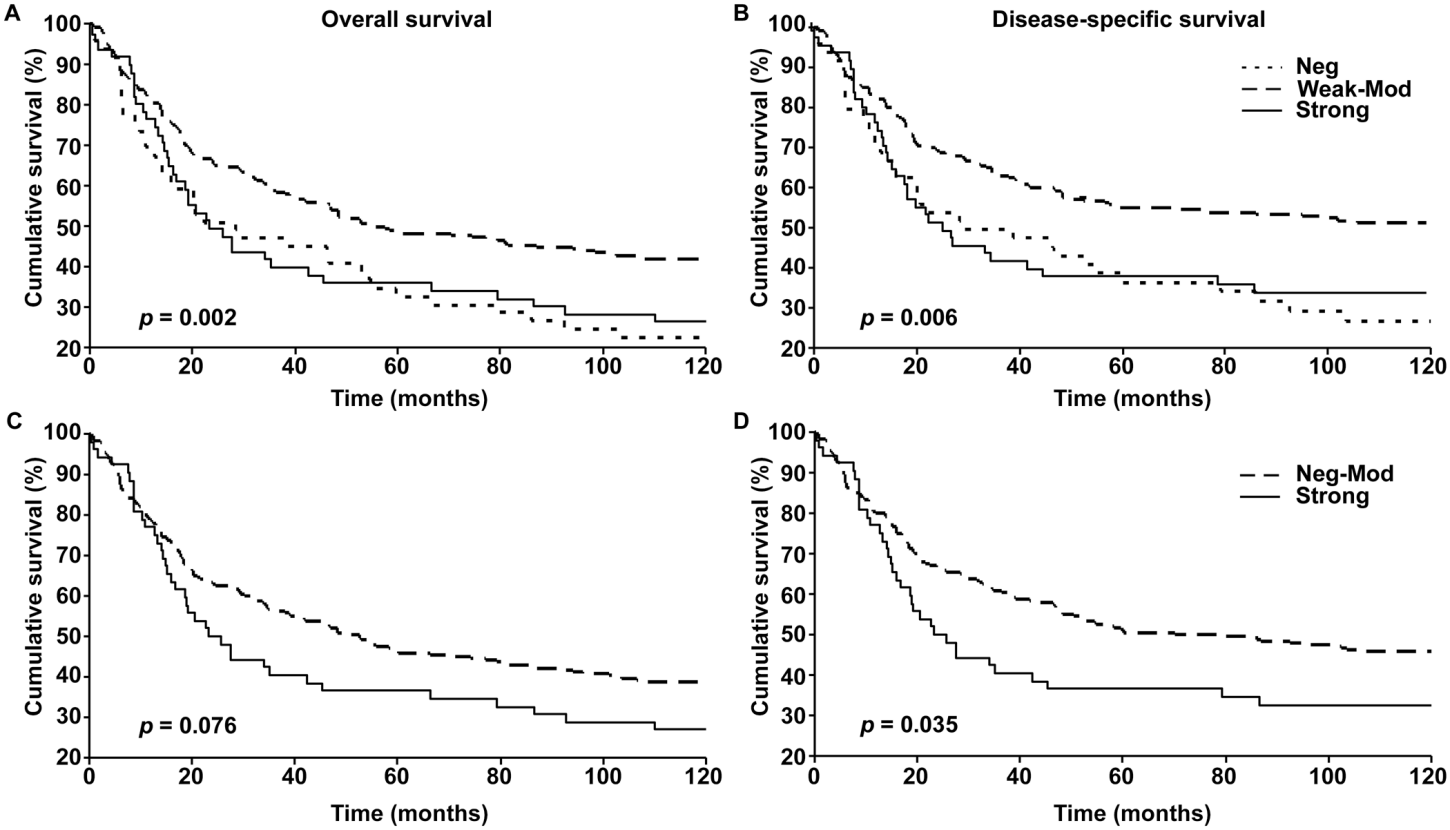
pATM expression and 10-year patient survival. Kaplan-Meier survival analyses of correlation between pATM expression and 10-year overall (left panels) and disease-specific (right panels) survival of melanoma patients classified as negative (Neg), weak-to-moderate (Weak-Mod) & strong (A and B), and as negative-to-moderate (Neg-Mod) & strong (C and D).

Next we analyzed the patient data where information on 10-year survival was available, to check if p-ATM had a similar association with 10-year patient survival. As seen with 5-year survival, patients with weak to moderate expression of p-ATM had better overall (p = 0.002) and disease specific (p = 0.006) 10-year survival compared to patients with strong p-ATM expression (Fig 4A and 4B). However, patients with negative p-ATM expression also had comparatively worse survival and its representative curve was found to almost overlap with that of the strong p-ATM expression (Fig 4A and 4B). When we combined the patients negative and weak to moderate p-ATM expression and compared the survival with patients with strong expression, the difference between the groups was slightly decreased as seen by increase in p-values. Strong p-ATM expression was found to be significantly associated with disease specific 10-year survival (Fig 4C and 4D). Similar results were obtained by univariate Cox regression analysis of the data as seen by significantly worse disease specific survival of patients with strong p-ATM expression compared to patients with negative to moderate expression of p-ATM (S2 Table).

### p-ATM expression is an independent prognostic factor for 5-year survival

Next we tested the prognostic significance of p-ATM expression by studying its ability to independently predict patient survival, using multivariate Cox regression analysis. We used p-ATM, age, AJCC stage and gender as a variables and analyzed the survival of the patients. p-ATM was categorized as negative to moderate vs strong, age as ≤ 60 vs > 60, AJCC as stages I & II vs stages III & IV, and gender as male vs female. As illustrated in Table 2, p-ATM was found to be significantly associated with 5-year survival, but not with 10-year survival of patients.

**Table 2:** Multivariate Cox regression analysis of overall and disease-specific survival in melanoma patients.

Variables*	Overall Survival				Disease-specific Survival			
	ß^†^	SE	HR (95% CI)	*p*-value	ß	SE	HR (95% CI)	*p*-value
**5-year**								
pATM	0.362	0.196	1.44 (0.98–2.11)	0.065	0.462	0.199	1.59 (1.07–2.34)	0.020
AJCC	1.313	0.156	3.72 (2.74–5.04)	3.4x10^-17^	1.504	0.167	4.50 (3.24–6.25)	2.5x10^-19^
Gender	0.286	0.157	1.33 (0.98–1.81)	0.069	0.279	0.166	1.32 (0.95–1.83)	0.093
Age	0.356	0.151	1.43 (1.06–1.92)	0.018	0.265	0.159	1.30 (0.95–1.78)	0.095
**10-year**								
pATM	0.314	0.182	1.37 (0.96–1.96)	0.085	0.370	0.192	1.45 (0.99–2.11)	0.054
AJCC	0.989	0.143	2.69 (2.03–3.56)	4.8x10^-12^	1.228	0.156	3.41 (2.51–4.64)	4.3x10^-15^
Gender	0.194	0.147	1.21 (0.91–1.62)	0.188	0.263	0.159	1.30 (0.95–1.78)	0.097
Age	0.457	0.141	1.58 (1.20–2.08)	0.001	0.311	0.152	1.37 (1.01–1.84)	0.040

*Coding of variables: Age was coded as 1 ((60 years), and 2 (>60 years). Gender was coded as 1 (male) and 2 (female). pATM expression was coded as 1 (neg-to-moderate) and 2 (strong). AJCC staging was coded as 1 (stages I & II), and 2 (stages III & IV).

^†^ ß: regression coefficient.

Abbreviations: SE, standard error of ß; HR, hazard ratio; CI, confidence interval.

## Discussion

Melanoma is one of the most commonly diagnosed cancers in fair skinned populations as shown by the recent report from the USA ranking it as the fifth most common cancer in men and seventh most common cancer in women [44]. Patients with advanced stages of melanoma have a very low survival rate (15%) and an overall survival duration between 8 to 18 months [44]. There are only a few treatment options available for metastatic melanoma due to its resistance to conventional chemo and radiotherapy [38–39]. The success of available treatment options is also limited with effectiveness restricted to a subset of patients, development of lethal resistance, or only a small increase in median survival time [38–39]. Consequently, the search for alternative drugs is ongoing.

Among the various origins of cancer, genomic instability is recognized as one of the major causes and UV radiation, a well-known genotoxic agent, is considered as one of the leading causes of melanoma [34, 45]. Previous studies identified that expression of several proteins involved in DNA repair were decreased or lost in melanoma patients, and that the loss of expression significantly correlated with poor patient survival [25–28, 31]. Our results here are partly in line with the previous data suggesting that a loss of mechanisms maintaining genomic integrity could be one of the critical events in melanoma pathogenesis.

The significance of ATM in cancer progression and patient prognosis has been shown in studies on tissue samples from gastric, pancreatic, bladder and endometrial cancers [19–20, 22, 46–48]. Previous studies on nodular metastatic melanoma samples showed an upregulation of ATM at both transcript and protein levels [24]. The sample size in that study was relatively low and the study did not report any association between ATM expression and patient survival. The study also did not analyze p-ATM expression in melanoma patients. Moreover, very few studies reported the relationship between p-ATM expression and patient survival [23]. Since ATM is a kinase, and its activation by phosphorylation at ser-1981 position allows it to associate itself with other DNA repair proteins as well as its down stream targets, analyzing p-ATM expression in patients could yield more accurate results [1, 3, 33, 40]. Accordingly, our findings illustrate an association between p-ATM expression, melanoma progression, and patient survival. ATM has important functions in DNA repair and maintenance of genomic integrity and understandably, loss of ATM could be associated with initiation and cancer progression [49]. On the other hand, ATM mediated regulation of signalling cascades could also drive cancer progression [49]. Our results showing that both loss of, and gain in, p-ATM expression are associated with melanoma progression points to the importance of tight regulation of cellular ATM levels. Strikingly, loss of p-ATM was seen to be more obviously associated with early stages of melanoma progression (stage I to stage II), and with tumor thickness, suggests a correlation between p-ATM expression and tumor growth. Our results are consistent with the reports from studies on tissue samples from pancreatic and gastric cancer patients, which showed that loss of ATM expression was associated with tumor progression [19–20, 47]. Previously, strong ATM expression was reported in nodular metastatic melanoma patients and our data showing a correlation between strong p-ATM expression and metastatic melanoma supports the hypothesis that ATM is involved in melanoma metastasis [24].

Along these lines, patients with strong p-ATM expression had the worst 5-year survival, patients with weak to moderate expression had the best survival, whereas patients with negative expression had intermediate survival. Understandably, a high level of activated p-ATM is associated with transformation of melanoma into a more aggressive and malignant phenotype and thereby associated with poor prognosis. Similarly, patients with negative expression of p-ATM might have had larger tumors, faster melanoma progression and consequently worse prognosis. The finding that patients with strong p-ATM had significantly worse survival compared to patients with negative to moderate expression, points to the oncogenic nature of ATM. The independence of strong p-ATM expression in predicting the survival outcome of patients in multivariate Cox regression analysis further supports its oncogenic nature. The therapeutic potential of ATM inhibition is currently being explored by researchers and ATM inhibitors have shown promise in preclinical xenograft models [50–52]. Our multivariate analysis data is also encouraging for additional research on the use of ATM inhibitors for treating melanoma. However, our results from 10-year survival analysis, showing close overlap of survival curves of patients with negative and strong p-ATM expression, points to the possible disadvantages of complete inhibition of the kinase.

Induction of apoptosis by DNA damaging agents like etoposide and cisplatin has been shown previously to cause caspase-3 mediated cleavage of ATM protein [53–54]. The cleaved ATM protein lacked the kinase activity against p53 but retained the DNA binding ability [53–54]. Our results on association between treatment with DNA damaging agents, chemoresistance and p-ATM expression indicates the critical role of ATM in cell survival and encourages further research on cleaved products of ATM.

Although our database was relatively large and we had information on most of the clinicopathologic characteristics of patients, due to the retrospective nature of the study we could not include all the recognized prognostic factors in our analysis. More studies with more patient information could conclusively determine the usefulness of ATM inhibition in the treatment of melanoma. Nevertheless, our study demonstrates association between p-ATM expression, melanoma progression and patient survival, and encourages further research. In summary, nuclear p-ATM expression has prognostic significance and may be a potential biomarker and molecular target for melanoma treatment.

## Supporting Information

S1 Fig pATM expression in nuclei of melanoma samples. (DOC)

S2 Fig Correlation between pATM expression and treatment with DNA damaging agents. (DOC)

S3 Fig Correlation between pATM expression and chemoresistance. (DOC)

S1 Table Demographics and clinical characteristics of 366 melanoma patients. (DOC)

S2 Table Univariate Cox regression analysis of overall and disease-specific survival in melanoma patients. (DOC)

## References

[1] Shiloh Y, Ziv Y. The ATM protein kinase: regulating the cellular response to genotoxic stress, and more. Nat Rev Mol Cell Biol. 2013;14(4):197–210. Epub 2013/07/13. . 23847781

[2] McKinnon PJ. ATM and ataxia telangiectasia. EMBO Rep. 2004;5(8):772–6. Epub 2004/08/04. 10.1038/sj.embor.7400210 7400210 [pii]. 15289825 PMC1299121

[3] Bakkenist CJ, Kastan MB. DNA damage activates ATM through intermolecular autophosphorylation and dimer dissociation. Nature. 2003;421(6922):499–506. Epub 2003/01/31. 10.1038/nature01368 nature01368 [pii]. . 12556884

[4] Smith GC, Cary RB, Lakin ND, Hann BC, Teo SH, Chen DJ, et al Purification and DNA binding properties of the ataxia-telangiectasia gene product ATM. Proc Natl Acad Sci U S A. 1999;96(20):11134–9. Epub 1999/09/29. 10500142 10.1073/pnas.96.20.11134 PMC17999

[5] Khoronenkova SV, Dianov GL. ATM prevents DSB formation by coordinating SSB repair and cell cycle progression. Proc Natl Acad Sci U S A. 2015;112(13):3997–4002. Epub 2015/03/17. 1416031112 [pii] 10.1073/pnas.1416031112 25775545 PMC4386361

[6] Wakasugi M, Sasaki T, Matsumoto M, Nagaoka M, Inoue K, Inobe M, et al Nucleotide excision repair-dependent DNA double-strand break formation and ATM signaling activation in mammalian quiescent cells. J Biol Chem. 2014;289(41):28730–7. Epub 2014/08/29. M114.589747 [pii] 10.1074/jbc.M114.589747 25164823 PMC4192521

[7] Chou WC, Hu LY, Hsiung CN, Shen CY. Initiation of the ATM-Chk2 DNA damage response through the base excision repair pathway. Carcinogenesis. 2015 Epub 2015/05/31. bgv079 [pii] 10.1093/carcin/bgv079 . 26025911

[8] Battu A, Ray A, Wani AA. ASF1A and ATM regulate H3K56-mediated cell-cycle checkpoint recovery in response to UV irradiation. Nucleic Acids Res. 2011;39(18):7931–45. Epub 2011/07/06. gkr523 [pii] 10.1093/nar/gkr523 21727091 PMC3185425

[9] Ray A, Milum K, Battu A, Wani G, Wani AA. NER initiation factors, DDB2 and XPC, regulate UV radiation response by recruiting ATR and ATM kinases to DNA damage sites. DNA Repair (Amst). 2013;12(4):273–83. Epub 2013/02/21. S1568-7864(13)00019-0 [pii] 10.1016/j.dnarep.2013.01.003 23422745 PMC4174315

[10] Savitsky K, Bar-Shira A, Gilad S, Rotman G, Ziv Y, Vanagaite L, et al A single ataxia telangiectasia gene with a product similar to PI-3 kinase. Science. 1995;268(5218):1749–53. Epub 1995/06/23. . 7792600 10.1126/science.7792600

[11] Perlman S, Becker-Catania S, Gatti RA. Ataxia-telangiectasia: diagnosis and treatment. Semin Pediatr Neurol. 2003;10(3):173–82. Epub 2003/12/05. . 14653405 10.1016/s1071-9091(03)00026-3

[12] Gao Y, Hayes RB, Huang WY, Caporaso NE, Burdette L, Yeager M, et al DNA repair gene polymorphisms and tobacco smoking in the risk for colorectal adenomas. Carcinogenesis. 2011;32(6):882–7. Epub 2011/04/21. bgr071 [pii] 10.1093/carcin/bgr071 21504893 PMC3106441

[13] Prokopcova J, Kleibl Z, Banwell CM, Pohlreich P. The role of ATM in breast cancer development. Breast Cancer Res Treat. 2007;104(2):121–8. Epub 2006/10/25. 10.1007/s10549-006-9406-6 . 17061036

[14] Rustgi AK. Familial pancreatic cancer: genetic advances. Genes Dev. 2014;28(1):1–7. Epub 2014/01/08. 28/1/1 [pii] 10.1101/gad.228452.113 24395243 PMC3894408

[15] Thorstenson YR, Roxas A, Kroiss R, Jenkins MA, Yu KM, Bachrich T, et al Contributions of ATM mutations to familial breast and ovarian cancer. Cancer Res. 2003;63(12):3325–33. Epub 2003/06/18. . 12810666

[16] Shen L, Yin Z, Wu W, Ren Y, Li X, Zhou B. Single Nucleotide Polymorphism in ATM Gene, Cooking Oil Fumes and Lung Adenocarcinoma Susceptibility in Chinese Female Non-Smokers: A Case-Control Study. PLoS One. 2014;9(5):e96911 Epub 2014/05/14. 10.1371/journal.pone.0096911 PONE-D-13-46027 [pii]. 24819391 PMC4018408

[17] Stankovic T, Kidd AM, Sutcliffe A, McGuire GM, Robinson P, Weber P, et al ATM mutations and phenotypes in ataxia-telangiectasia families in the British Isles: expression of mutant ATM and the risk of leukemia, lymphoma, and breast cancer. Am J Hum Genet. 1998;62(2):334–45. Epub 1998/04/16. S0002-9297(07)63499-5 [pii] 10.1086/301706 9463314 PMC1376883

[18] Kim H, Saka B, Knight S, Borges M, Childs E, Klein A, et al Having pancreatic cancer with tumoral loss of ATM and normal TP53 protein expression is associated with a poorer prognosis. Clin Cancer Res. 2014;20(7):1865–72. Epub 2014/02/04. 1078-0432.CCR-13-1239 [pii] 10.1158/1078-0432.CCR-13-1239 24486587 PMC3975663

[19] Yu G, Zhu MH, Zhu Z, Ni CR, Zheng JM, Li FM. Expression of ATM protein and its relationship with p53 in pancreatic carcinoma with tissue array. Pancreas. 2004;28(4):421–6. Epub 2004/04/21. 00006676-200405000-00011 [pii]. . 15097860 10.1097/00006676-200405000-00011

[20] Kang B, Guo RF, Tan XH, Zhao M, Tang ZB, Lu YY. Expression status of ataxia-telangiectasia-mutated gene correlated with prognosis in advanced gastric cancer. Mutat Res. 2008;638(1–2):17–25. Epub 2007/10/12. S0027-5107(07)00323-5 [pii] 10.1016/j.mrfmmm.2007.08.013 . 17928013

[21] Rondeau S, Vacher S, De Koning L, Briaux A, Schnitzler A, Chemlali W, et al ATM has a major role in the double-strand break repair pathway dysregulation in sporadic breast carcinomas and is an independent prognostic marker at both mRNA and protein levels. Br J Cancer. 2015;112(6):1059–66. Epub 2015/03/06. bjc201560 [pii] 10.1038/bjc.2015.60 25742469 PMC4366900

[22] Mhawech-Fauceglia P, Wang D, Kim G, Sharifian M, Chen X, Liu Q, et al Expression of DNA repair proteins in endometrial cancer predicts disease outcome. Gynecol Oncol. 2014;132(3):593–8. Epub 2014/02/11. S0090-8258(14)00120-6 [pii] 10.1016/j.ygyno.2014.02.002 . 24508840

[23] Roossink F, Wieringa HW, Noordhuis MG, ten Hoor KA, Kok M, Slagter-Menkema L, et al The role of ATM and 53BP1 as predictive markers in cervical cancer. Int J Cancer. 2012;131(9):2056–66. Epub 2012/02/11. 10.1002/ijc.27488 22323184 PMC3504092

[24] Roesch A, Becker B, Bentink S, Spang R, Vogl A, Hagen I, et al Ataxia telangiectasia-mutated gene is a possible biomarker for discrimination of infiltrative deep penetrating nevi and metastatic vertical growth phase melanoma. Cancer Epidemiol Biomarkers Prev. 2007;16(11):2486–90. Epub 2007/11/17. 16/11/2486 [pii] 10.1158/1055-9965.EPI-07-0224 . 18006941

[25] Bhandaru M, Ardekani GS, Zhang G, Martinka M, McElwee KJ, Li G, et al A combination of p300 and Braf expression in the diagnosis and prognosis of melanoma. BMC Cancer. 2014;14(1):398 Epub 2014/06/05. 1471-2407-14-398 [pii] 10.1186/1471-2407-14-398 . 24893747 PMC4051389

[26] Bhandaru M, Martinka M, Li G, Rotte A. Loss of XRCC1 confers a metastatic phenotype to melanoma cells and is associated with poor survival in patients with melanoma. Pigment Cell Melanoma Res. 2014;27(3):366–75. Epub 2014/01/15. 10.1111/pcmr.12212 . 24410901

[27] Rotte A, Bhandaru M, Cheng Y, Sjoestroem C, Martinka M, Li G. Decreased expression of nuclear p300 is associated with disease progression and worse prognosis of melanoma patients. PLoS One. 2013;8(9):e75405 Epub 2013/10/08. 10.1371/journal.pone.0075405 PONE-D-13-24955 [pii]. 24098694 PMC3787094

[28] Chen G, Cheng Y, Tang Y, Martinka M, Li G. Role of Tip60 in human melanoma cell migration, metastasis, and patient survival. J Invest Dermatol. 2012;132(11):2632–41. Epub 2012/06/08. jid2012193 [pii] 10.1038/jid.2012.193 . 22673729

[29] Wong RP, Aguissa-Toure AH, Wani AA, Khosravi S, Martinka M, Li G. Elevated expression of Rad18 regulates melanoma cell proliferation. Pigment Cell Melanoma Res. 2012;25(2):213–8. Epub 2011/12/08. 10.1111/j.1755-148X.2011.00948.x . 22145991

[30] Zhang G, Li G. Novel multiple markers to distinguish melanoma from dysplastic nevi. PLoS One. 2012;7(9):e45037 Epub 2012/10/03. 10.1371/journal.pone.0045037 PONE-D-12-16762 [pii]. 23028750 PMC3459895

[31] Lin H, Wong RP, Martinka M, Li G. Loss of SNF5 expression correlates with poor patient survival in melanoma. Clin Cancer Res. 2009;15(20):6404–11. Epub 2009/10/08. 1078-0432.CCR-09-1135 [pii] 10.1158/1078-0432.CCR-09-1135 . 19808872

[32] Chen G, Lee E. The product of the ATM gene is a 370-kDa nuclear phosphoprotein. J Biol Chem. 1996;271(52):33693–7. Epub 1996/12/27. . 8969240 10.1074/jbc.271.52.33693

[33] Guo Z, Kozlov S, Lavin MF, Person MD, Paull TT. ATM activation by oxidative stress. Science. 2010;330(6003):517–21. Epub 2010/10/23. 330/6003/517 [pii] 10.1126/science.1192912 . 20966255

[34] Gonzaga ER. Role of UV light in photodamage, skin aging, and skin cancer: importance of photoprotection. Am J Clin Dermatol. 2009;10 Suppl 1:19–24. Epub 2009/01/01. 1014 [pii]. 10.2165/0128071-200910001-00004 19209950

[35] Zhang Z, Chen G, Cheng Y, Martinka M, Li G. Prognostic significance of RUNX3 expression in human melanoma. Cancer. 2011;117(12):2719–27. Epub 2011/06/10. 10.1002/cncr.25838 . 21656750

[36] Remmele W, Stegner HE. [Recommendation for uniform definition of an immunoreactive score (IRS) for immunohistochemical estrogen receptor detection (ER-ICA) in breast cancer tissue]. Pathologe. 1987;8(3):138–40. Epub 1987/05/01. . 3303008

[37] Camp RL, Dolled-Filhart M, Rimm DL. X-tile: a new bio-informatics tool for biomarker assessment and outcome-based cut-point optimization. Clin Cancer Res. 2004;10(21):7252–9. Epub 2004/11/10. 10/21/7252 [pii] 10.1158/1078-0432.CCR-04-0713 . 15534099

[38] Cheng Y, Zhang G, Li G. Targeting MAPK pathway in melanoma therapy. Cancer Metastasis Rev. 2013;32(3–4):567–84. Epub 2013/04/16. 10.1007/s10555-013-9433-9 . 23584575

[39] Rotte A, Bhandaru M, Zhou Y, McElwee KJ. Immunotherapy of melanoma: present options and future promises. Cancer Metastasis Rev. 2015;34(1):115–28. Epub 2015/01/16. 10.1007/s10555-014-9542-0 . 25589384

[40] Lee JH, Paull TT. Activation and regulation of ATM kinase activity in response to DNA double-strand breaks. Oncogene. 2007;26(56):7741–8. Epub 2007/12/11. 1210872 [pii] 10.1038/sj.onc.1210872 . 18066086

[41] Moschos SJ, Dodd NR, Jukic DM, Fayewicz SL, Wang X, Becker D. Suppressing the high-level expression and function of ATM in advanced-stage melanomas does not sensitize the cells to ionizing radiation. Cancer Biol Ther. 2009;8(19):1815–25. Epub 2009/08/18. 9435 [pii]. . 19684476 10.4161/cbt.8.19.9435

[42] Eich M, Roos WP, Nikolova T, Kaina B. Contribution of ATM and ATR to the resistance of glioblastoma and malignant melanoma cells to the methylating anticancer drug temozolomide. Mol Cancer Ther. 2013;12(11):2529–40. Epub 2013/08/21. 1535-7163.MCT-13-0136 [pii] 10.1158/1535-7163.MCT-13-0136 . 23960094

[43] Ivanov VN, Zhou H, Partridge MA, Hei TK. Inhibition of ataxia telangiectasia mutated kinase activity enhances TRAIL-mediated apoptosis in human melanoma cells. Cancer Res. 2009;69(8):3510–9. Epub 2009/04/09. 0008-5472.CAN-08-3883 [pii] 10.1158/0008-5472.CAN-08-3883 19351839 PMC4070218

[44] Siegel R, Naishadham D, Jemal A. Cancer statistics, 2013. CA Cancer J Clin. 2013;63(1):11–30. Epub 2013/01/22. 10.3322/caac.21166 . 23335087

[45] Peifer M, Fernandez-Cuesta L, Sos ML, George J, Seidel D, Kasper LH, et al Integrative genome analyses identify key somatic driver mutations of small-cell lung cancer. Nat Genet. 2012;44(10):1104–10. Epub 2012/09/04. ng.2396 [pii] 10.1038/ng.2396 . 22941188 PMC4915822

[46] Kim HS, Kim MA, Hodgson D, Harbron C, Wellings R, O'Connor MJ, et al Concordance of ATM (ataxia telangiectasia mutated) immunohistochemistry between biopsy or metastatic tumor samples and primary tumors in gastric cancer patients. Pathobiology. 2013;80(3):127–37. Epub 2013/01/19. 000346034 [pii] 10.1159/000346034 . 23328638

[47] Lee HE, Han N, Kim MA, Lee HS, Yang HK, Lee BL, et al DNA damage response-related proteins in gastric cancer: ATM, Chk2 and p53 expression and their prognostic value. Pathobiology. 2014;81(1):25–35. Epub 2013/08/24. 000351072 [pii] 10.1159/000351072 . 23969480

[48] Shin JU, Lee CH, Lee KT, Lee JK, Lee KH, Kim KM, et al Prognostic significance of ATM and cyclin B1 in pancreatic neuroendocrine tumor. Tumour Biol. 2012;33(5):1645–51. Epub 2012/06/19. 10.1007/s13277-012-0420-5 . 22707287

[49] Stagni V, Oropallo V, Fianco G, Antonelli M, Cina I, Barila D. Tug of war between survival and death: exploring ATM function in cancer. Int J Mol Sci. 2014;15(4):5388–409. Epub 2014/04/01. ijms15045388 [pii] 10.3390/ijms15045388 24681585 PMC4013570

[50] Biddlestone-Thorpe L, Sajjad M, Rosenberg E, Beckta JM, Valerie NC, Tokarz M, et al ATM kinase inhibition preferentially sensitizes p53-mutant glioma to ionizing radiation. Clin Cancer Res. 2013;19(12):3189–200. Epub 2013/04/27. 1078-0432.CCR-12-3408 [pii] 10.1158/1078-0432.CCR-12-3408 23620409 PMC3687028

[51] Batey MA, Zhao Y, Kyle S, Richardson C, Slade A, Martin NM, et al Preclinical evaluation of a novel ATM inhibitor, KU59403, in vitro and in vivo in p53 functional and dysfunctional models of human cancer. Mol Cancer Ther. 2013;12(6):959–67. Epub 2013/03/21. 1535-7163.MCT-12-0707 [pii] 10.1158/1535-7163.MCT-12-0707 23512991 PMC3736091

[52] Khalil HS, Tummala H, Hupp TR, Zhelev N. Pharmacological inhibition of ATM by KU55933 stimulates ATM transcription. Exp Biol Med (Maywood). 2012;237(6):622–34. Epub 2012/06/26. ebm.2012.011378 [pii] 10.1258/ebm.2012.011378 . 22728709

[53] Smith GC, d'Adda di Fagagna F, Lakin ND, Jackson SP. Cleavage and inactivation of ATM during apoptosis. Mol Cell Biol. 1999;19(9):6076–84. Epub 1999/08/24. 10454555 10.1128/mcb.19.9.6076 PMC84521

[54] Wang J, Pabla N, Wang CY, Wang W, Schoenlein PV, Dong Z. Caspase-mediated cleavage of ATM during cisplatin-induced tubular cell apoptosis: inactivation of its kinase activity toward p53. Am J Physiol Renal Physiol. 2006;291(6):F1300–7. Epub 2006/07/20. 00509.2005 [pii] 10.1152/ajprenal.00509.2005 . 16849690

