# Supplementary figures and images for: Prognostic Significance of Nuclear Phospho-ATM Expression in Melanoma
Source: PLoS One. 2015 Aug 14;10(8):e0134678. doi: 10.1371/journal.pone.0134678 (PMC4537129; doi:10.1371/journal.pone.0134678)

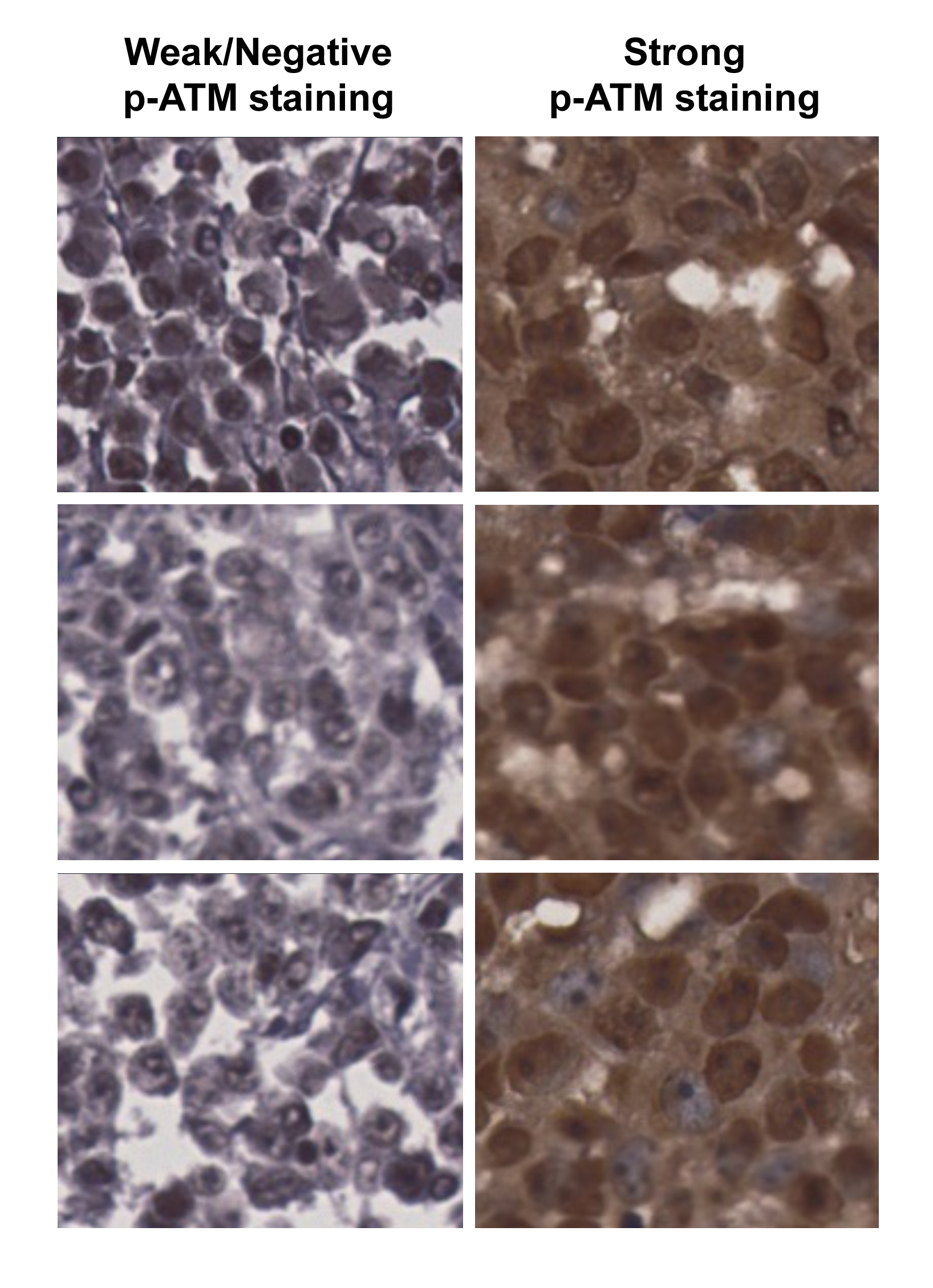


S1 Fig. Representative images of p-ATM staining at higher magnification (800x)

Supplement: S1 Fig — (DOC) [file pone.0134678.s001.doc]

**
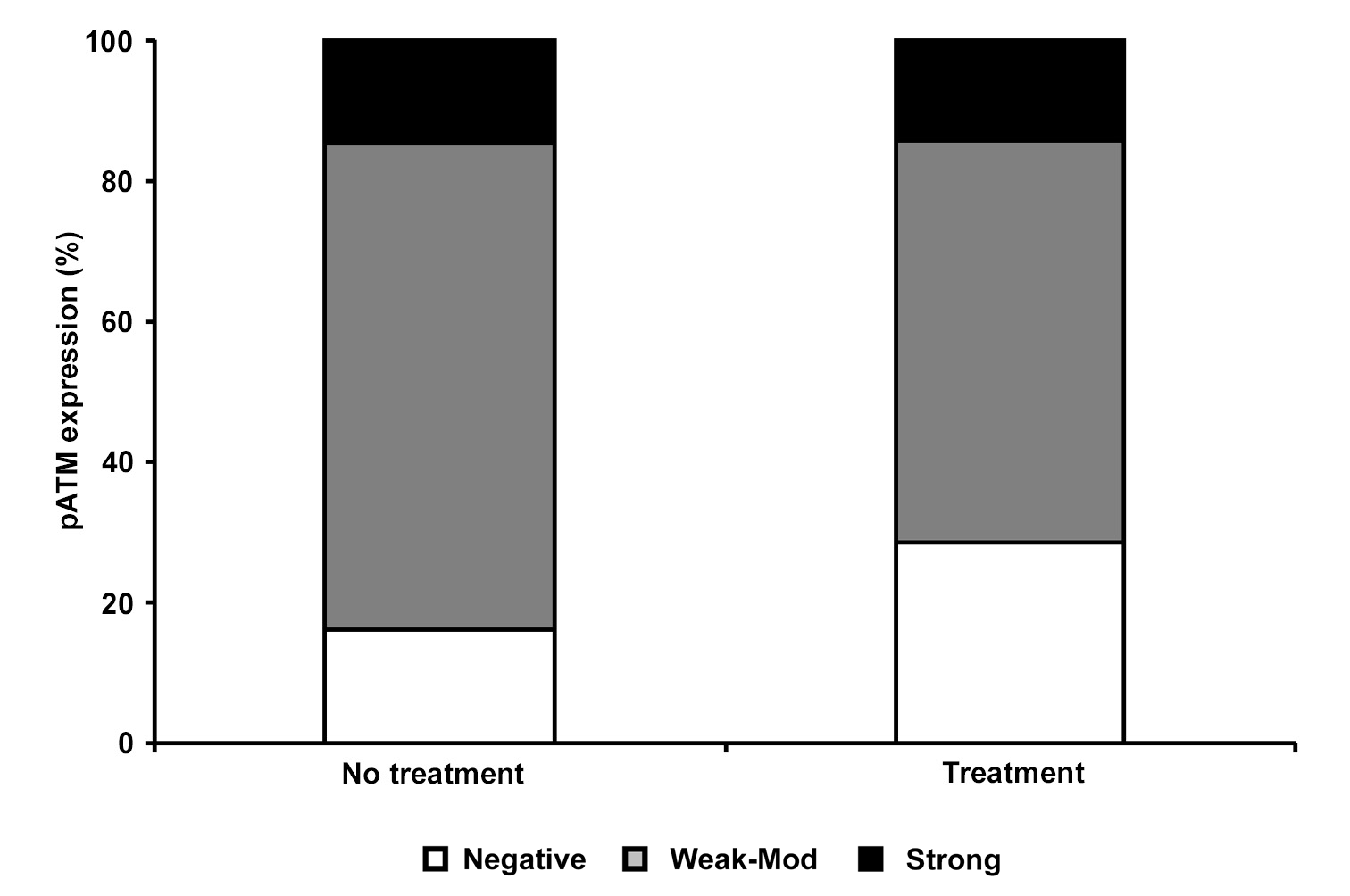
**

S2 Fig. Correlation between pATM expression and treatment with DNA damaging agents

p=0.328 (χ2 test)

Supplement: S2 Fig — (DOC) [file pone.0134678.s002.doc]
