# Supplementary material for: Prognostic Significance of Nuclear Phospho-ATM Expression in Melanoma
Source: PLoS One. 2015 Aug 14;10(8):e0134678. doi: 10.1371/journal.pone.0134678 (PMC4537129; doi:10.1371/journal.pone.0134678)
Supplement: S3 Fig — (DOC) [file pone.0134678.s003.doc]

**
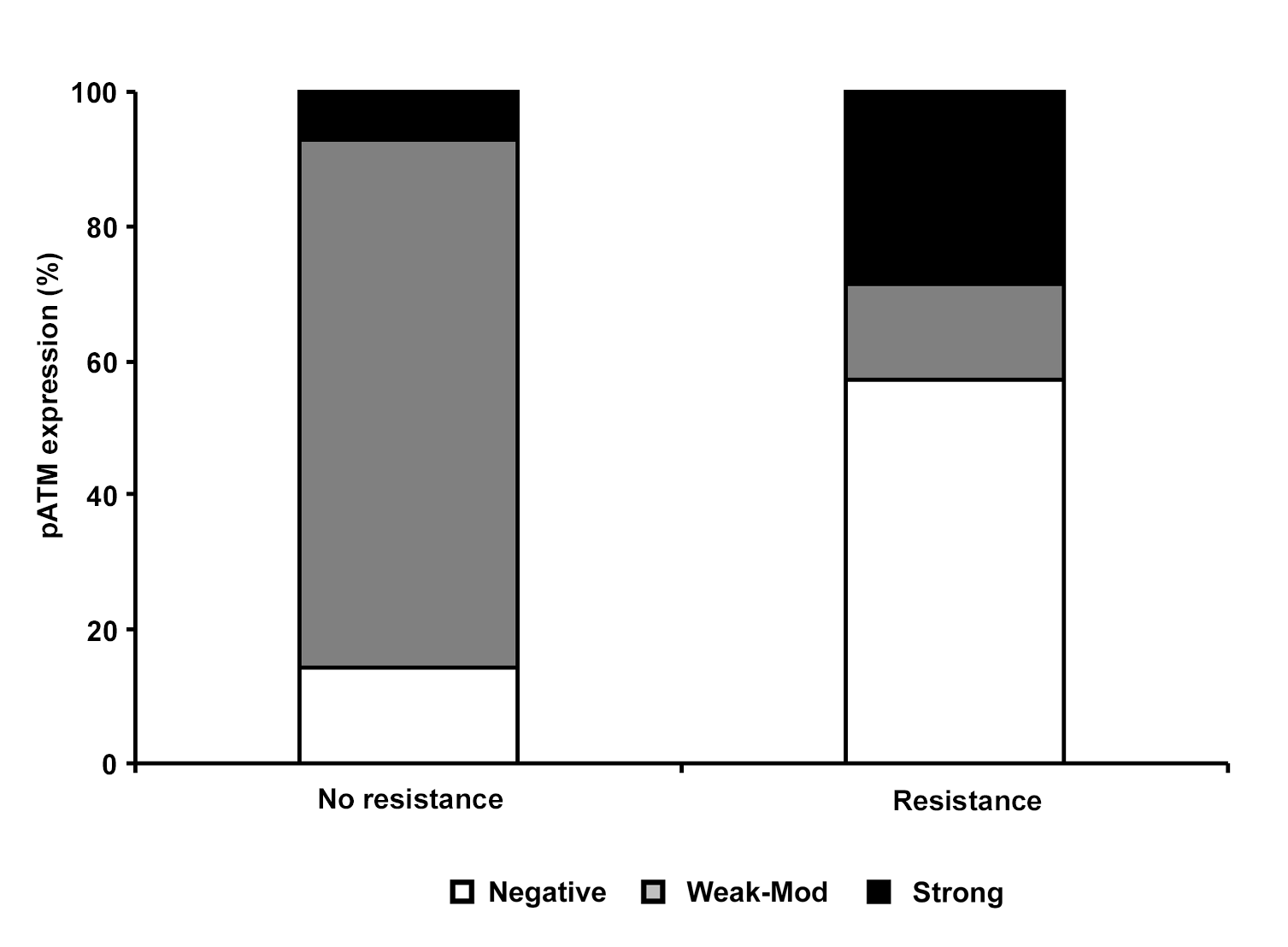
**

S3 Fig. Correlation between pATM expression and resistance to treatment with DNA damaging agents

p=0.0194 (χ^2^ test)
