# Supplementary material for: Prognostic Significance of Nuclear Phospho-ATM Expression in Melanoma
Source: PLoS One. 2015 Aug 14;10(8):e0134678. doi: 10.1371/journal.pone.0134678 (PMC4537129; doi:10.1371/journal.pone.0134678)
Supplement: S1 Table — (DOC) [file pone.0134678.s004.doc]

**S1 Table.** Demographics and clinical characteristics of 366 melanoma patients

| Variables | Total | Percentage |
| --- | --- | --- |
| All melanoma |  |  |
| Age  ≤ 60 | 189 | 51.6% |
| > 60 | 177 | 48.4% |
| Gender  Male | 219 | 59.8% |
| Female | 147 | 40.2% |
| AJCC  I | 102 | 27.9% |
| II | 128 | 35.0% |
| III | 55 | 15.0% |
| IV | 81 | 22.1% |
| Site  Sun Protected | 281 | 76.8% |
| Sun Exposed | 85 | 23.2% |
| Primary melanoma (n = 230) |  |  |
| Age  ≤ 60 | 110 | 47.8% |
| > 60 | 120 | 52.2% |
| Gender  Male | 127 | 55.2% |
| Female | 103 | 44.8% |
| Thickness  ≤ 2.0 mm | 114 | 49.6% |
| > 2.0 mm | 116 | 50.4% |
| Ulceration  Absent | 117 | 77.0% |
| Present | 53 | 23.0% |
| Subtype  Acrolentigous | 8 | 3.5% |
| Lentigous | 34 | 14.8% |
| Nodular | 45 | 19.6% |
| Spindle cell | 10 | 4.3% |
| Superficially spreading | 78 | 33.9% |
| Unspecified | 55 | 23.9% |
| Metastatic melanoma (n = 136) |  |  |
| Age  ≤ 60 | 79 | 58.1% |
| > 60 | 57 | 41.9% |
| Gender  Male | 92 | 67.6% |
| Female | 44 | 32.4% |
