# Supplementary material for: Prognostic Significance of Nuclear Phospho-ATM Expression in Melanoma
Source: PLoS One. 2015 Aug 14;10(8):e0134678. doi: 10.1371/journal.pone.0134678 (PMC4537129; doi:10.1371/journal.pone.0134678)
Supplement: S2 Table — (DOC) [file pone.0134678.s005.doc]

**S2 Table.** Univariate Cox regression analysis of overall and disease-specific survival in melanoma patients.

| Variables | **Overall Survival** | | | |  | **Disease-specific Survival** | | | |
| --- | --- | --- | --- | --- | --- | --- | --- | --- | --- |
|  | ß^†^ | SE | HR (95 % CI) | *p*-value |  | ß | SE | HR (95 % CI) | *p*-value |
| **5-year** | | | | | | | | | |
| pATM | 0.437 | 0.193 | 1.55 (1.06-2.26) | 0.024 |  | 0.566 | 0.196 | 1.76 (1.20-2.59) | 0.004 |
| AJCC | 1.261 | 0.152 | 3.53 (2.62-4.76) | 1.3x10^-16^ |  | 1.465 | 0.164 | 4.33 (3.14-5.97) | 3.5x10^-19^ |
| Gender | 0.010 | 0.152 | 1.01 (0.75-1.36) | 0.945 |  | -0.036 | 0.162 | 0.96 (0.70-1.32) | 0.821 |
| Age | 0.241 | 0.150 | 1.27 (0.95-1.71) | 0.107 |  | 0.129 | 0.158 | 1.14 (0.84-1.55) | 0.412 |
| **10-year** | | | | | | | | | |
| pATM | 0.318 | 0.180 | 1.37 (0.97-1.96) | 0.077 |  | 0.397 | 0.189 | 1.49 (1.03-2.15) | 0.036 |
| AJCC | 0.962 | 0.141 | 2.62 (1.98-3.45) | 1.0x10^-11^ |  | 1.191 | 0.154 | 3.29 (2.43-4.45) | 1.1x10^-14^ |
| Gender | -0.004 | 0.144 | 1.00 (0.75-1.32) | 0.978 |  | 0.022 | 0.155 | 1.02 (0.75-1.39) | 0.885 |
| Age | 0.411 | 0.141 | 1.51 (1.14-1.99) | 0.003 |  | 0.242 | 0.151 | 1.27 (0.95-1.71) | 0.109 |

*Coding of variables: Age was coded as 1 ((60 years), and 2 (>60 years). Gender was coded as 1 (male) and 2 (female). pATM expression was coded as 1 (neg-to-moderate) and 2 (strong). AJCC staging was coded as 1 (stages I & II), and 2 (stages III & IV). † ß: regression coefficient.

Abbreviations: SE, standard error of ß; HR, hazard ratio; CI, confidence interval.
